# The Penguin Study: A Randomised, Double-Blinded, Equivalence Trial on the Safety and Suitability of an Infant Formula with Partially Hydrolysed 100% Whey Protein

**DOI:** 10.3390/pediatric17020045

**Published:** 2025-04-09

**Authors:** Lindsey Otten, Antonia Nomayo, Caroline Gunn, Maher Fuad, Barbara Kuhn-Sherlock, Sophie Gallier, Elisabeth Schelker, Janine Foster, Frank Jochum

**Affiliations:** 1 Department of Pediatrics, Evangelisches Waldkrankenhaus Spandau, Stadtrandstraße 555, 13589 Berlin, Germany; 2 Fonterra Co-Operative Group Limited, Palmerston North 4442, New Zealand; 3 BKS Statistical Consulting, Hamilton 3216, New Zealand; 4 Sophie Gallier Science Consulting, Hamilton 3200, New Zealand; 5 Brandenburg Medical School Theodor Fontane (MHB), Fehrbelliner Str. 38, 16816 Neuruppin, Germany

**Keywords:** hydrolysate, hydrolysed infant formula, infant nutrition, neonate, nutrition, infant growth, infant weight gain, breastfeeding

## Abstract

**Background/Objectives:** This study aimed to demonstrate the safety and suitability of an infant formula manufactured from partially hydrolysed whey protein (PHF) compared to standard formula manufactured from intact cow’s milk proteins (IPF; whey–casein ratio, 60:40) in healthy term infants. **Methods:** This multicentre, randomised, double-blinded, placebo-controlled trial included infants of mothers who intended to exclusively formula feed. Infants ≤ 28 days of age received PHF or IPF for at least 90 and up to 180 days. A group of exclusively breastfed infants was included for reference. The safety evaluation consisted of an equivalence analysis of weight gain within +/−3 g/d after 90 days, further growth parameters, and adverse events. **Results:** Of the 249 infants randomised, 143 (76 IPF; 67 PHF), as well as 45 breastfed infants, completed the study per protocol. The mean difference in daily weight gain between the formula groups was within the equivalence margins (−2.4 g/d (95% CI 0.3–4.5)) with estimated means (SEM) of 34.9 (0.78) g/d (IPF) and 32.5 (0.76) g/d (PHF). No significant differences in weight gain, length, and head circumference or in the number, severity, or type of adverse events were observed. Comparable growth patterns were observed in the breastfed group. **Conclusions:** The PHF is safe and supports adequate infant growth with a daily weight gain non-inferior to a standard IPF.

## 1. Introduction

Breastfeeding is the ideal and recommended form of nutrition for infants; however, if (exclusive) breastfeeding is not possible or desired, infant formula is available. Starter infant formulae must fulfil the nutritional requirements to promote adequate growth and development of term infants when used as the sole source of nutrition during the first months of life, as well as when used as the principal liquid element upon introduction of complementary feeding (Commission Directive 2006/141/EC of 22 December 2006). In addition to standard infant formulae manufactured from intact cow’s milk protein, infant formulae manufactured from partially or extensively hydrolysed cow’s milk protein are widely available. While the latter have, in the past, been recommended in expert guidelines as a measure of allergy prevention or to improve functional gastrointestinal symptoms [1,2,3], scientific evidence remains limited [3,4,5].

Regardless of functional benefit, it must be demonstrated that the modified protein structure in infant formula manufactured from a hydrolysed protein source leads to non-inferior infant growth and development compared to formula with intact protein. The European Commission (EC) requires that the safety and suitability of each particular infant formula containing protein hydrolysate is demonstrated through clinical trials [6].

The present study aimed to demonstrate the safety and suitability of a specific infant formula with partially hydrolysed 100% whey protein (PHF) compared to an infant formula containing intact cow’s milk protein (IPF) with the same macronutrient and micronutrient composition. The primary objective was to evaluate the equivalence between the two products with respect to daily weight gain as the main indicator of healthy growth in healthy term infants over a 90-day period of exclusive feeding of the investigational formulae. Secondary objectives included further growth parameters measured up to 180 days of age. A group of non-randomised, healthy, term, exclusively breastfed infants was included as a reference.

## 2. Materials and Methods

### 2.1. Study Centres

Thirteen paediatric departments of hospitals in Germany and three paediatric departments of hospitals in Bulgaria participated in the study from November 2018 to January 2021. The study was conducted under consideration of ICH Good Clinical Practice (GCP, E6 (R2), November 2016) and the Declaration of Helsinki—version 2013, and in accordance with the local legal and regulatory requirements. It was approved by the corresponding ethics committees and entered prospectively in the German Clinical Trials Register (DRKS; DRKS00016462).

### 2.2. Study Design and Population

This study was a multicentre, randomised, controlled, double-blinded equivalence trial of two parallel groups. A non-randomised reference group of healthy, term, breastfed infants was also included. The total study duration was 6 months; however, the primary intervention period, in which equivalence in mean daily weight gain between the two formulae was evaluated, was 90 days from the day of enrolment, i.e., 90 study days. The secondary objectives, including further growth parameters, were evaluated over the entire 180-day feeding period or until 6 months of age. Eligible infants were enrolled up to 28 days of age. After the enrolment visit (visit 1 (V1), 0–28 days of age), infants and mothers attended study visits after 30 ± 3 (visit 2 (V2)), 60 ± 3 (visit 3 (V3)) and 90 ± 7 (visit 4 (V4)) study days, and at 6 months of age ±7 days (visit 5 (V5)). A three-day diary, including formula intake and gastrointestinal and behavioural parameters, was to be filled out by the parents during the three days prior to each visit.

Healthy, term infants from singleton pregnancies and with a gestational age between ≥37 weeks + 0 days and ≤41 weeks + 6 days, birth weight between 2500 and 4500 g, and APGAR score ≥ 7 (10 min after birth), as well as the mother’s intention to exclusive formula feeding from the time of enrolment to at least 90 study days thereafter were eligible for study participation. Exclusion criteria included: adverse maternal, foetal, or infant medical history that may have potential effects on tolerance, growth, and/or development of the infant, insulin-dependent gestational diabetes, allergy to cow’s milk proteins or increased risk of allergy, rehospitalisation within 5 days of birth, infants receiving medication that might affect gastrointestinal tolerance (including antibiotic therapy), participation in another clinical trial or the intention to use a combination of breast and formula feeding. Complementary food and drink were permitted after V4 in accordance with national and international recommendations [2,7].

Mothers of suitable neonates for the formula groups were only addressed if they were not able to or for personal reasons would not be breastfeeding, despite advice on the advantages of breastfeeding.

Upon written informed consent of the parents, eligible infants were enrolled in the study and randomised in a ratio of 1:1 to receive the test formula (Partially Hydrolysed Formula (PHF): infant formula with 100% whey protein hydrolysate) or the control formula (Intact Protein Formula (IPF): standard infant formula; whey–casein ratio of 60:40).

Infants who met the above criteria and whose mothers intended to exclusively breastfeed for at least 90 days were included in the reference breastfed group. Recruitment in the breastfed group was based on each hospital site’s intervention recruitment rate at a ratio of 4:1 (intervention–breastfed) in order to achieve a similar distribution of breastfed infants across the study centres and recruitment period compared to the formula-fed infants.

Study arm allocation was conducted in a centralised manner. The computer-generated randomisation lists were stratified by site and gender in blocks of ten and provided in sealed, consecutively numbered envelopes to each study site. All screened subjects were given a unique subject identification number. Identification numbers assigned to non-eligible subjects and prematurely discontinued subjects were not re-used.

The manufacturer blinded the two formulae using a three-digit code that did not allow the deduction of the formula identity. Only the manufacturer, with no contact with the clinical study team, was unblinded to the identity of the tin contents. The products were indistinguishable by smell and appearance. The blinding of formulae was concealed until the completion of the study.

### 2.3. Study Product

The two formula products used in the study had the same macro- and micronutrient composition with vitamins and minerals in the amount intended for exclusive feeding of infants aged 0–6 months (Table 1). Both products were manufactured by Hochdorf Swiss Nutrition AG (Hochdorf, Switzerland) and complied with Regulation (EU) 2016/127 on the specific compositional requirements for infant formula [6,8]. The only difference between the products was the replacement of the whey and casein proteins in the IPF (whey–casein ratio, 60:40) with 100% partially hydrolysed whey proteins (NZMP WPH7051 sourced from Fonterra (Auckland, New Zealand)) in the PHF. The protein content was 1.3 g/100 mL or 2.0 g/100 kcal.

### 2.4. Anthropometric Measurements

Measures of body weight, length, and head circumference were taken at each visit. Weight and length were measured using an electronic scale with an integrated, analogue measuring rod. Head circumference was measured using a head circumference measuring tape.

Due to the coronavirus pandemic, alterations in the study protocol had to be made. To ensure the safety of study personnel and participants, personal contact was reduced as much as possible by performing study visits (except the enrolment visit) by telephone and allowing parents to perform the anthropometric measurements themselves at home from May 2020 to the end of the study in January 2021. Additionally, in Bulgaria, the outpatient care units of the study sites were used for study visits when pandemic restrictions allowed it. To maintain a high level of data quality, parents were schooled in measuring techniques during the enrolment visit at the study clinic and provided with quality measuring equipment (portable electronic infant scale, ADE M112600, max. 20 kg ± 5 g, ADE Germany GmbH & Co. KG, Hamburg, Germany; infant measuring mat, ADE MZ10027-1, 100–990 mm ± 5 mm, ADE Germany GmbH & Co. KG; head circumference measuring tape, seca 212, 1–59 cm ± 0.01 cm, seca GmbH & Co., Hamburg, Germany).

### 2.5. Formula Intake

Formula intake (number of feedings per day, consumed volume at each feeding, and any complementary food or drink provided) was documented in diaries completed by the parents during the three days prior to each visit (starting from V2).

In addition to documenting formula intake, parents were asked to return the empty cans of formula to the clinic in order to assess compliance.

### 2.6. Overall Health and Adverse Events

At each visit, the infant’s parents were asked about signs of gastrointestinal tolerance, allergy, and infection since the previous visit, including bloating/flatulence, constipation, diarrhoea, spitting up, vomiting, diaper rash and colic, wheezing, rash or eczema, fever (>38.5 degrees Celsius), coughing, runny or stuffy nose, conjunctivitis, otitis or others. Such symptoms were also to be noted in the 3-day diaries. Furthermore, all medications were documented, including the reason for intake, dose, and duration of use.

Adverse and serious adverse events (AEs, SAEs) were documented throughout the study. Investigators followed up on (S)AEs until they were resolved.

### 2.7. Statistics

Safety was primarily monitored and assessed as a measure of equivalence in daily weight gain between the PHF and IPF (active comparator) over a period of 90 days. The pre-specified equivalence margin of −3 g/day to +3 g/day was based on the recommendation from the American Academy of Paediatrics [9].

Both intention-to-treat (ITT) and per-protocol (PP) analyses were planned to be performed. The ITT population included all subjects who were randomised to a study group and received the control or test infant formulae. The PP analysis set included all subjects randomised into the study and attending all visits, with no major protocol violations and adequate compliance. While the PP set was the main focus for analyses of the primary objective (i.e., infant weight gain after a 90-day exclusive feeding period (V1 to V4) of the PHF compared to IPF), results from the ITT population were also taken into consideration. For the analyses of the secondary objectives, the results from PP up to V5 are presented below; the results from ITT up to V5 are presented in the Supplementary Materials.

Participants were considered non-compliant if they did not attend all visits, were fed non-exclusively, i.e., were fed more than one bottle of another formula per week up to V4; did not participate in the study for a minimum of 3 consecutive days; or were given a complementary food before V4.

The sample size calculation for an equivalence trial of two infant formulae was based on the primary outcome of average daily weight gain, targeting 90% power and 5% significance and assuming a standard deviation (SD) of 6 g/d [10,11]. Accordingly, 86 subjects per group were required to detect a difference of 3 g/d. To account for 30% attrition, the target was to recruit 120 subjects for each infant formula group. In the end, the study achieved 80% power (lower than the targeted 90%) because the number of PP subjects was smaller than expected (67 and 76) and the SD greater than assumed (6.4 g/d).

The statistical analyses were performed using SAS 9.4 (2016, SAS Institute Inc., Cary, NC, USA). Significance was declared if *p* ≤ 0.05. Data were log_10_-transformed if necessary to achieve homogeneity of variance.

Baseline data are presented as mean ± SD (continuous variables) and count and percentages (categorical variables).

Average daily weight gain (g/day) was calculated as the weight gain between V1 and V4 divided by the number of days between V1 and V4 and was analysed using an analysis of covariance (ANCOVA) model. The model included the infant formula group (IPF or PHF), country, and their interaction as fixed effects, sex as a fixed blocking factor, and gestational age and weight at V1 as covariates. Results are presented as least-squares means (LSM) and standard error of the mean (SEM), mean difference, and 95% confidence interval (95% CI) between interventions, as well as the partial eta squared value (Partial η^2^).

Growth variables (absolute and z-scores) and formula intake at each visit were analysed up to V5 using a mixed model’s approach to repeated measures analysis of variance (ANOVA). The models included visit, infant formula group, country, and their interactions as fixed effects, sex (growth variables and formula intake only) as a fixed blocking factor, gestational age as a covariate, and infant as a random factor. The covariance pattern chosen was first-order autoregressive. ANOVA was followed by post-hoc tests using Tukey adjustment for multiple comparisons in order to test for differences between study arms at each visit. Results are presented as LSM and SEM.

The incidence of adverse events was analysed using binary logistic regression with country, sex, and study arm included as fixed effects. Results are presented as counts.

## 3. Results

### 3.1. Study Population Characteristics

**Figure 1:**
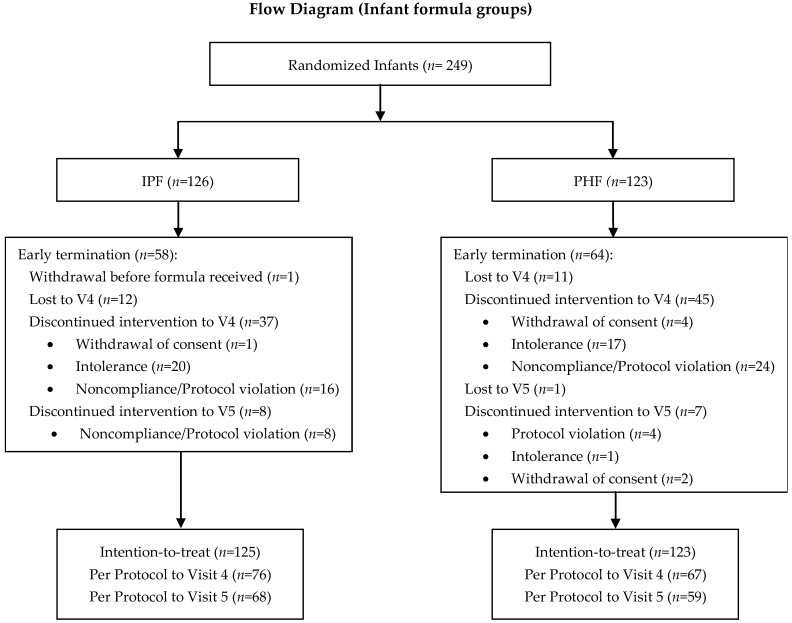
Flow Diagram of Infant Formula Groups. IPF: infant formula manufactured from intact cow’s milk proteins; n: number of observations; PHF: infant formula manufactured from partially hydrolysed whey protein. V4: 90 ± 7 study days; V5: 6 months ± 7 study days.

Two hundred and forty-nine healthy infants were randomised into one of the two infant formula groups (126 in IPF; 123 in PHF), and 49 infants were included in the non-randomised breastfed reference group. A total of 143 (63%) randomised infants (76 in IPF; 67 in PHF) and 45 (92%) breastfed infants completed the study per protocol (PP) up to 90 days (V4). Early termination of study procedures to V4 and, therefore, inclusion only in the ITT analysis did not differ between the two formula groups (n = 49/125 (39%) vs. n = 56/123 (46%)). Reasons for early termination largely included lost to follow-up and discontinued intervention due, for example, to intolerance/adverse events and non-compliance with regard to feeding or visit dates (Figure 1). One participant randomised to receive IPF withdrew consent before receiving any study product and was not included in the data analysis. In the breastfed group, two infants discontinued participation after V1, and two were fed formula in addition to breastmilk and were, therefore, not included in the PP analysis.

The characteristics of the PP to V4 population, on which the primary objective is based, are presented in Table 2. There were no differences in birth or parental characteristics or in anthropometric parameters at birth or study baseline between the formula groups (Table 2). Infants in the breastfed group were less often born by caesarean section. At birth and study baseline, infants in the breastfed group had slightly higher birth weight and length than infants in the formula groups. Mothers and fathers of infants in the breastfed group had a lower BMI than those in the formula groups.

### 3.2. Growth

During the coronavirus pandemic, all baseline anthropometric measurements were performed at the study sites, both in Germany and Bulgaria. In Germany, three V4 measurements were performed by a midwife or paediatrician, and 24 V4 and 34 V5 visits were performed by parents at home with the help of written instructions. In Bulgaria, six V4 measurements were performed by parents at home under telephone assistance and/or written instructions; all other visits were performed at the study sites. Statistical analysis revealed no significant interaction of measurement method, i.e., at home versus at the study site, with the formula groups; therefore, the measurement method was removed from the model.

#### 3.2.1. Primary Objective: Equivalence in Weight Gain

The difference in estimated mean daily weight gain (g/d) from study baseline (V1) to 90 days (V4) between the formula groups was 2.4 g/d (95% CI (0.3–4.5); Table 3, “All”). Mean daily weight gain was significantly higher in PHF than IPF in the PP but not the ITT population. The model showed a significant effect of country in the PP and of sex in both the PP and ITT populations. Due to the significant effect of country, the presented data are separated by country (Table 3). There was no interaction, however, between the formula group and country in the PP (*p* = 0.163) or ITT (*p* = 0.256) populations. The results show a trend for lower mean daily weight gain in the IPF group in Bulgaria compared to the other groups. In all cases, the effect size was small to medium.

#### 3.2.2. Secondary Objectives: Further Growth Parameters

The analyses of absolute weight, length, and head circumference in the PP (Table 4) and ITT (Table S1) populations showed no significant effect of the intervention over the entire duration of the study. Age (i.e., visit number) had a significant effect (*p* < 0.001) in the subset analyses due to the infants’ growth during the intervention period. In addition, sex and country had a significant influence on growth.

Infants in Germany had lower absolute mean weight (5499.2 ± 46.2 vs. 5530.7 ± 53.8 g (*p* < 0.001)) and greater mean length (58.8 ± 0.21 vs. 57.0 ± 0.24 g (*p* = 0.003)) and head circumference (39.1 ± 0.10 vs. 38.4 ± 0.12 g (*p* = 0.023)) than in Bulgaria in the ITT population. The results remained significant (*p* < 0.001) for length and head circumference in the PP population.

**Figure 2:**
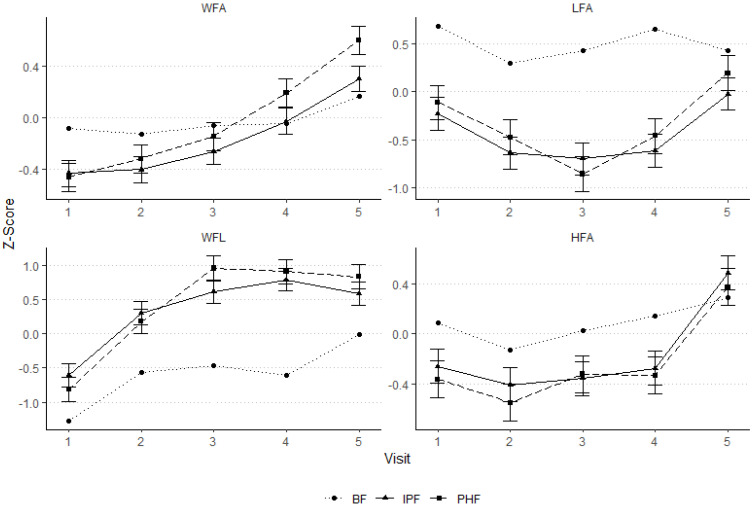
Z-Scores for weight-for-age (WFA), length-for-age (LFA), weight-for-length (WFL), and head circumference-for-age (HFA) at visits 1 to 5 for IPF and PHF in the PP population. Each symbol represents the least square mean (LSM) +/−1 × standard error of the mean (SEM). For reference, the breastfed group is shown with raw means as well. Based on WHO child growth standards [12]. IPF: infant formula manufactured from intact cow’s milk proteins; n: number of observations; PHF: infant formula manufactured from partially hydrolysed whey protein; PP: per-protocol population; WHO: World Health Organization. V1: 0–28 days of age; V2: 30 ± 3 study days; V3: 60 ± 3 study days; V4: 90 ± 7 study days; V5: 6 months ± 7 study days.

Compared to the WHO reference populations [12], z-scores for weight-for-age (WFA), length-for-age (LFA), weight-for-length (WFL), and head circumference-for-age (HFA) showed no significant effect of the intervention, i.e., no differences between IPF and PHF, in PP (Figure 2) or ITT (Figure S1) populations. All values for both formula groups were within the range of ±1 SD. Consistent with the results above, country had an influence (*p* < 0.05) on all z-scores in PP and ITT. The WFA-, LFA-, WFL-, and HFA-z-scores for Germany and Bulgaria separately are shown in Figures S2 and S3, respectively. The z-scores for the breastfed group are included for reference.

### 3.3. Formula Intake

Formula consumption did not differ between the formula groups (Table 5 and Table S2). Average daily intake volume per kilogram body weight was significantly associated with the duration of the intervention (*p* < 0.001) and did not differ between countries (*p* = 0.589). The average number of daily feedings recorded was higher in the breastfed compared to formula groups at all visits (mean (SD) for PP at V5: breastfed 6.54 (1.919) vs. IPF 4.79 (0.933) and PHF 4.53 (1.160)).

### 3.4. Behaviour and Tolerance

Aspects of behaviour such as crying episodes, waking at night, or perceived irritability were documented in the 3-day diaries before each visit. Further, parents were asked if they had noticed any particular issues or problems which could be linked to formula tolerance, such as constipation, diarrhoea, reflux or vomiting, and infant colic, at each study visit.

In addition to the above-mentioned tolerance-related symptoms, parents reported symptoms, including flatulence, watery stool, stomach pain, and nappy rash, as reasons for study discontinuation (Figure 1).

Symptoms mentioned at each visit and as a reason for study discontinuation did not differ between the formula groups.

### 3.5. Adverse Events

There was no difference in the number of infants with at least one adverse event (AE) throughout the entire duration of the trial to V5 (IPF 75/125 vs. PHF 65/123, (*p* = 0.256); breastfed 35/49). The number of events classified as severe was not significantly different either (IPF 10/152 vs. PHF 9/111 (*p* = 0.636); breastfed 7/80). All of the seven recorded serious adverse events were unrelated to the intervention used.

## 4. Discussion

In this prospective, multicentre, randomised, controlled, double-blinded trial, infants who received the formula manufactured from partially hydrolysed 100% whey protein (PHF) experienced non-inferior weight gain to infants who received the control formula manufactured from intact cow’s milk proteins (IPF). Furthermore, growth with respect to weight, length, and head circumference were within the expected ranges for full-term healthy infants [12], and no safety concerns arose based on the frequency and type of (serious) adverse events. We conclude that the PHF used in this trial can be considered safe for use in healthy-term infants.

The mean difference (PHF–IPF) in estimated daily weight gain of 2.4 (95% CI: 0.3–4.5) g/day in the PP population was within the equivalence margin of ±3 g/day. In both the PP and ITT populations, the upper 95% CI crossed the upper equivalence bound of +3 g/day; therefore, while not equivalent, PHF can be considered non-inferior to IPF. While mean daily weight gain was statistically higher in infants who received PHF in the PP, this was not the case in the ITT population. However, comparable studies investigating partially hydrolysed 100% whey protein infant formula have reported equal weight gain between the PHF and IPF the intact protein formula (no differences in weight gain (g/d) [13]; −0.076 (90% CI: −1.252; 1.100) g/day [14]; −0.474 (95% CI: −2.460, 1.512) g/day [15]) or a tendency to inferior weight gain in the PHF group (Picaud et al., 2020: −1.2 (90% CI: −2.42; 0.02) g/d) [16]. In general, weight differences between formula groups were smaller in previous studies, and absolute weight gains were lower. Infants receiving the PHF in this study experienced mean daily weight gain of 34.9 (SEM 0.78) g/day, while infants in the trials from Karaglani et al., Rigo et al., Picaud et al. and Kantaras et al. reported means of 24.06 (SEM 2.64) g/day [15], 29.6 (SD 5.8) g/day [17], 30.2 (SEM 0.5) [16], and 30.9 (95% CI: 29–8; 31.9) g/day [14], respectively, for partially hydrolysed formula-fed groups.

Note that these studies vary somewhat in age at baseline and study duration. With respect to the study from Karaglani et al. [15], for example, infants were much older at the start of the intervention (55–80 days) compared to the present and other referenced studies. This may have had an impact on the difference in the rate of daily weight gain between the groups. Nevertheless, post-hoc analyses were performed in order to investigate the origin of the relatively large margin in our study. It became clear that the mean daily weight gain in IPF in Bulgaria strongly deviated from PHF in Bulgaria as well as IPF and PHF in Germany. A closer examination of formula intake between the countries and groups showed no differences. Additionally, there were no differences in product batches between the two countries. Despite the observed variation in weight, it can be noted that the effect size shown in the statistical analyses was small to medium; therefore, the clinical significance may be negligible.

Cultural differences and feeding patterns in the differing countries may be one factor contributing to the variance in reported weight gain among studies. This aspect is supported by the fact that the statistical analyses in the present study showed no significant intervention effects for any of the subset analyses. The studies referenced above included countries in Europe. The present study included participants from Germany and Bulgaria in equal proportions.

With respect to feeding patterns, some studies have reported a difference in formula intake between the intact and partially hydrolysed formula-fed groups and attributed this to negative sensory characteristics of hydrolysate formula, such as bitter taste [15,18]. As discussed by Mennella et al., these negative sensory characteristics are first recognised as such by infants after a period of flavour programming, and when infants receive the hydrolysate formula early on, it is generally well accepted [18,19]. Correspondingly, other studies, including the present study, in which infants received the formula within the first days or weeks of life, have shown no difference in intake of intact compared to hydrolysed protein formula [16]. The consumed volume corrected for body weight as well as the energy density (kcal/100 mL) were comparable between the present study and that of Picaud et al. The macronutrient composition of the two formulae in this study is also comparable to those used by Picaud et al. [16].

The exact molecular weight of the proteins in the various hydrolysates is usually unknown [3]. Various studies have shown comparable weight gain in infants who received intact, partially hydrolysed, or extensively hydrolysed protein formula [10,20], suggesting the little impact of the degree of hydrolysis on weight gain.

While the gestational age of both formula-fed and breastfed infants was comparable, formula-fed infants were more often born by caesarean section, and mothers of infants in the formula-fed groups had a higher BMI. There is a known association between BMI and delivery mode [21] as well as BMI and cessation of exclusive breastfeeding [22,23]; however, this aspect was not further evaluated in this study. Compared to the breastfed group, infants in the formula-fed groups had a slightly lower weight at birth, which increased and was comparable throughout the rest of the study period, and lower body length throughout the study period, as reflected in the z-scores (observational comparison; not statistically tested). Changes in body weight and length in breastfed compared to formula-fed infants in previous studies vary. While some studies link differences in growth between breastfed and formula-fed infants to differences in protein intake [24], Botton et al. described the influence of maternal and paternal BMI and height on early childhood growth [25]; parental BMI and height varied somewhat between the groups in our study and may therefore partially explain the variation in body weight and length in breastfed and formula-fed infants. Consistent with previous findings, breastfed infants in this trial had a higher feeding frequency than formula-fed infants (descriptive) [26,27].

This trial was conducted during the peak of the COVID pandemic with accompanying government advisories, including compulsory face masks, restrictions on mobility, and lockdowns, as well as media coverage of the virus spread, cases, and deaths. It is likely this may have impacted parents’ stress levels, which may, in turn, have contributed to the relatively high dropout rate. Notably, the most common reasons for early study termination or non-inclusion in the per-protocol evaluation were formula intolerance, as evaluated by the parents, and non-compliance with regard to visit dates and feeding requirements. Interestingly, there was a low dropout rate in the breastfed group. Despite the infant’s weight gains, growth, and health being within normal parameters, any longer-term impact of the pandemic situation on the infant’s well-being is still to be elucidated.

This trial was required to guarantee the safety of infant formula as a sole nutrient source for infants and has been presented to EFSA for evaluation of PHF. The obtained scientific data on growth patterns of exclusively breastfed or formula-fed infants in two different countries can aid in the further development of nutritional products that are closer to the gold standard of breastmilk for infants who are not breastfed.

In summary, it was shown that PHF is safe and suitable for exclusive feeding of infants in their first four months of life.

## Figures and Tables

**Table 1:** Nutritional composition of intervention products.

Content per 100 mL	PHF	IPF
Energy (kcal/kJ)	64/269	64/269
Fat (g) *	3.26	3.26
Saturated (g)	1.4	1.4
Monounsaturated (g)	1.2	1.2
Polyunsaturated (g)	0.59	0.59
Linoleic acid (mg)	422.4	422.4
Alpha linolenic acid (mg)	57.0	57.0
Arachidonic acid (mg)	15.4	15.4
Docosahexaenoic acid (mg)	15.4	15.4
Protein (g)	1.3	1.3
Whey–Casein	100:0	60:40
Carbohydrate (g) ^†^	7.28	7.28
scGOS (g)	0.54	0.54

IPF: infant formula manufactured from intact cow’s milk proteins; scGOS: short chain oligosaccharides; PHF: infant formula manufactured from partially hydrolysed whey protein; * Sources of fat: Anhydrous milk fat and vegetable oil (50:50). ^†^ Sources of total carbohydrates: Lactose and scGOS.

**Table 2:** Demographic and clinical characteristics of the PP population to V4.

Parameter	IPF (n = 76)	PHF (n = 67)	Breastfed (n = 45)
Sex, girls	35 (46.1)	33 (49.3)	24 (53.3)
Birth characteristics			
Caesarean section	38 (50.7)	40 (59.7)	17 (38.6)
Complications	4 (5.3)	0 (0)	0 (0)
Gestational age (weeks)	38.8 ± 1.02	38.9 ± 0.99	39.0 ± 1.04
Weight (g)	3285.2 ± 448.74	3278.8 ± 381.85	3421.9 ± 346.18
Length (cm)	50.3 ± 2.14	50.6 ± 2.30	51.5 ± 2.05
Head circumference (cm)	34.7 ± 1.42	34.5 ± 1.33	34.7 ± 1.34
Baseline characteristics			
Age (days)	9.3 ± 9.21	8.9 ± 8.38	7.9 ± 6.78
Weight (g)	3339.7 ± 501.79	3303.8 ± 457.00	3433.9 ± 382.42
Length (cm)	50.6 ± 2.14	50.8 ± 2.18	52.1 ± 2.22
Head circumference (cm)	34.9 ± 1.48	34.7 ± 1.30	35.0 ± 1.38
Parental characteristics			
Weight of mother (kg)	75.1 ± 17.11	74.8 ± 14.18	68.6 ± 15.32
Height of mother (cm)	165.3 ± 6.95	166.4 ± 6.77	166.3 ± 6.33
BMI of mother (kg/m^2^)	27.5 ± 6.16	27.0 ± 5.26	24.8 ± 5.16
Age of mother (years)	30.5 ± 5.65	29.7 ± 5.84	31.9 ± 4.42
Weight of father (kg)	88.4 ± 15.47	90.2 ± 17.44	86.5 ± 10.79
Height of father (cm)	178.7 ± 6.09	177.8 ± 7.70	181.0 ± 7.15
BMI of father (kg/m^2^)	27.6 ± 4.36	28.5 ± 5.14	26.4 ± 3.23
Age of father (years)	34.0 ± 7.02	33.2 ± 7.68	34.2 ± 4.78
Country			
Germany	40 (52.6)	34 (50.8)	21 (46.7)
Bulgaria	36 (47.4)	33 (49.3)	24 (53.3)

Values are presented as total numbers and percentages [n (%)] or mean ± SD. BMI: body mass index; IPF: infant formula manufactured from intact cow’s milk proteins; n: number of observations; PHF: infant formula manufactured from partially hydrolysed whey protein; PP: per-protocol population.

**Table 3:** Mean daily weight gain in the formula groups from V1 to V4.

Population	Country	Group	N	Weight Gain (g/d) V1 to V4. LSM (SEM)	Difference Between Groups. Estimate (95% CI)	Sex *p*-Value	Country *p*-Value	Group *p*-Value
						Partial η^2^	Partial η^2^	Partial η^2^
PP	All	PHF	67	34.9 (0.77)	2.4 (0.3–4.5)	0.002	0.038	0.027
		IPF	76	32.5 (0.73)		0.07	0.03	0.04
	Germany	PHF	34	35.3 (1.10)	0.9 (−2.0–3.9)			
		IPF	40	34.4 (1.02)				
	Bulgaria	PHF	33	34.5 (1.11)	3.9 (0.9–7.0)			
		IPF	36	30.5 (1.08)				
ITT	All	PHF	123	34.6 (0.70)	1.9 (−0.0–3.9)	<0.001	0.092	0.059
		IPF	125	32.7 (0.69)		0.08	0.02	0.02
	Germany	PHF	76	35.0 (1.00)	0.79 (−1.91–3.49)			
		IPF	75	34.2 (0.96)				
	Bulgaria	PHF	47	34.3 (1.01)	3.05 (0.23–5.86)			
		IPF	50	31.2 (1.02)				

Gestational age and weight at V1 were also included in the model but were not significant (*p* > 0.05). All: refers to both countries combined; CI: confidence interval; η^2^: eta squared; IPF: infant formula manufactured from intact cow’s milk proteins; ITT: intention-to-treat population; LSM: least square mean; n: number of observations; PHF: infant formula manufactured from partially hydrolysed whey protein; PP: per-protocol population; SEM: standard error of the mean. V1: visit 1, 0–28 days of age; V4: visit 4, 90 ± 7 study days.

**Table 4:** Repeated measures ANOVA for absolute growth parameters of the PP population to V5.

Variable	Visit	IPF (n = 68)		PHF (n = 59)		Breastfed (n = 45)
		LSM	SEM	LSM	SEM	Mean
Weight (g) ^a,c,e,f^	V1	3340.9	76.36	3284.8	81.27	3433.9
	V2	4421.4		4438.3		4506.6
	V3	5434.0		5491.7		5496.8
	V4	6305.0		6447.0		6232.7
	V5	7920.0		8207.9		7714.4
Length (cm) ^a,b,c,e^	V1	50.6	0.34	50.7	0.37	52.1
	V2	54.0		54.2		55.6
	V3	57.2		56.8		59.3
	V4	60.0		60.3		62.5
	V5	66.6		67.1		67.3
Head circumference (cm) ^a,b,e^	V1	34.8	0.17	34.6	0.18	35.0
	V2	37.0		36.8		37.2
	V3	38.7		38.7		39.0
	V4	40.0		39.9		40.4
	V5	43.4		43.2		43.0

Values are presented as least-squares means (LSM) and standard error of the mean (SEM) for formula groups. Significant effect (*p* < 0.05): ^(a)^ sex; ^(b)^ country; ^(c)^ gestational age; ^(e)^ visit; ^(f)^ intervention × visit. The breastfed group is included for reference only; values are presented as raw means. ANOVA: analysis of variance; IPF: infant formula manufactured from intact cow’s milk proteins; n: number of observations; PHF: infant formula manufactured from partially hydrolysed whey protein; PP: per-protocol population. V1: 0–28 days of age; V2: 30 ± 3 study days; V3: 60 ± 3 study days; V4: 90 ± 7 study days; V5: 6 months ± 7 study days.

**Table 5:** Formula intake relative to body weight (mL/kg/day) in PP to V5.

Visit	IPF			PHF			*p*-Value
	N	LSM	SEM	N	LSM	SEM	
V2	63	161.6	3.60	58	162.8	3.78	0.823
V3	63	143.51	3.59	58	144.5	3.78	0.845
V4	67	129.0	3.49	54	132.7	3.88	0.475
V5	65	101.8	3.54	56	94.5	3.88	0.164

Values are presented as least square mean (LSM) and standard error of the mean (SEM). V1: 0–28 days of age; V2: 30 ± 3 study days; V3: 60 ± 3 study days; V4: 90 ± 7 study days; V5: 6 months ± 7 study days.

## Data Availability

Restrictions apply to the availability of these data. Data may be made available to certain parties for specified purposes (e.g., meta-analysis) with the permission of the study sponsors Fonterra Co-operative Group Limited, Hero AG, Hochdorf Swiss Nutrition AG, DMK Baby GmbH, and Ausnutria (Hyproca).

## Supplementary Materials

The following supporting information can be downloaded at https://www.mdpi.com/article/10.3390/pediatric17020045/s1. Figure S1: Z-Scores for weight-for-age (WFA), length-for-age (LFA), weight-for-length (WFL), and head circumference-for-age (HFA) for visits V1 to V5 for IPF and PHF in the ITT population; Figure S2: Z-Scores for weight-for-age (WFA), length-for-age (LFA), weight-for-length (WFL) and head circumference-for-age (HFA) for visits V1 to V5 for IPF and PHF in the ITT population in Germany; Figure S3: Z-Scores for weight-for-age (WFA), length-for-age (LFA), weight-for-length (WFL) and head circumference-for-age (HFA) for visits V1 to V5 for IPF and PHF in the ITT population in Bulgaria; Table S1: Repeated measures ANOVA for absolute growth parameters of the ITT population to V5; Table S2: Formula intake relative to body weight (mL/kg/day) in ITT to V5.
