# Supplementary material for: The Penguin Study: A Randomised, Double-Blinded, Equivalence Trial on the Safety and Suitability of an Infant Formula with Partially Hydrolysed 100% Whey Protein
Source: Pediatr Rep. 2025 Apr 9;17(2):45. doi: 10.3390/pediatric17020045 (PMC12030296; doi:10.3390/pediatric17020045)
Supplement: Supplementary file 1 [file pediatrrep-17-00045-s001.zip › pediatrrep-3454563-supplementary.pdf]

Supplementary Materials

Table S1. Repeated measures ANOVA for absolute growth parameters of the ITT population to V5.

Variable	Visit	IPF		PHF		Breastfed	
		LSM	SEM	LSM	SEM	n	Mean
Weight (g)[a, b, c, e, g]	V1 (n=244)	3363.5	54.45	3314.5	55.45	48	3433.2
	V2 (n=187)	4425.0	56.52	4444.0	58.11	47	4517.8
	V3 (n=178)	5435.1	58.51	5479.7	60.17	47	5506.1
	V4 (n=168)	6302.3	60.52	6414.3	62.07	47	6235.3
	V5 (n=159)	7867.4	61.97	8104.0	64.53	47	7733.3
Length (cm)[a, b, c, e]	V1 (n=245)	50.8	0.26	50.8	0.26	48	52.1
	V2 (n=187)	54.1	0.27	54.3	0.28	47	55.6
	V3 (n=178)	57.2	0.28	57.3	0.29	47	59.3
	V4 (n=168)	60.3	0.29	60.6	0.30	47	62.5
	V5 (n=160)	66.5	0.30	67.1	0.31	47	67.5
Head circumference (cm)[a, b, c, e]	V1 (n=242)	34.9	0.14	34.8	0.14	48	35.1
	V2 (n=186)	36.9	0.14	36.9	0.15	47	37.3
	V3 (n=176)	38.7	0.15	38.7	0.16	46	39.1
	V4 (n=166)	40.1	0.16	40.0	0.16	47	40.5
	V5 (n=160)	43.4	0.16	43.1	0.17	47	43.1

Values are presented as least-squares means (LSM) and standard error of the mean (SEM) for formula groups. Significant effect ($p<0.05$): a) sex; b) country; c) gestational age; e) visit; g) intervention*country*visit. The breastfed group is included for reference only; values are presented as raw means.
ANOVA: analysis of variance; IPF: infant formula manufactured from intact cow's milk proteins; ITT: intention-to-treat population; n: number of observations; PHF: infant formula manufactured from partially hydrolysed whey protein.
V1: 0-28 days of age; V2: 30 ± 3 study days; V3: 60 ± 3 study days; V4: 90 ± 7 study days; V5: 6 months ± 7 study days.

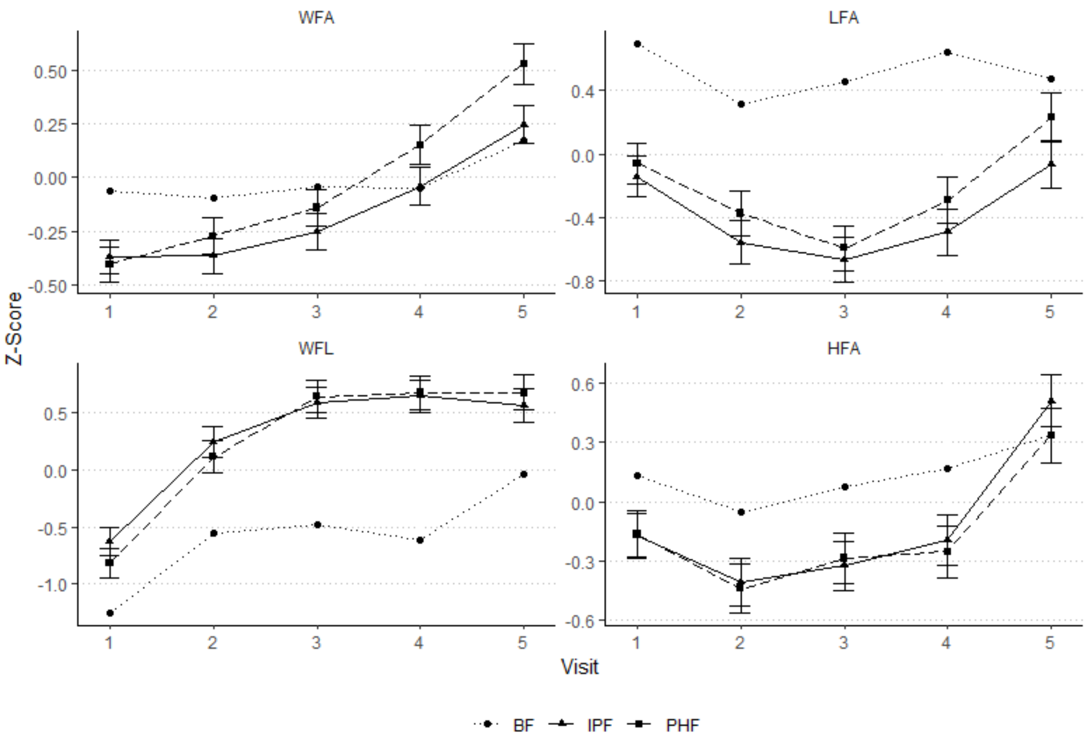

Figure S1. Z-Scores for weight-for-age (WFA), length-for-age (LFA), weight-for-length (WFL), and head circumference-for-age (HFA) for visits V1 to V5 for IPF and PHF in the ITT population. Each symbol represents the least square mean (LSM) +/- 1 x standard error of the mean (SEM). For reference, the breastfed group is shown with raw means. Based on WHO child growth standards [18].

IPF: infant formula manufactured from intact cow's milk proteins; ITT: intention-to-treat population; n: number of observations; PHF: infant formula manufactured from partially hydrolysed whey protein; WHO: World Health Organization.

V1: 0-28 days of age; V2: 30 ± 3 study days; V3: 60 ± 3 study days; V4: 90 ± 7 study days; V5: 6 months ± 7 study days.

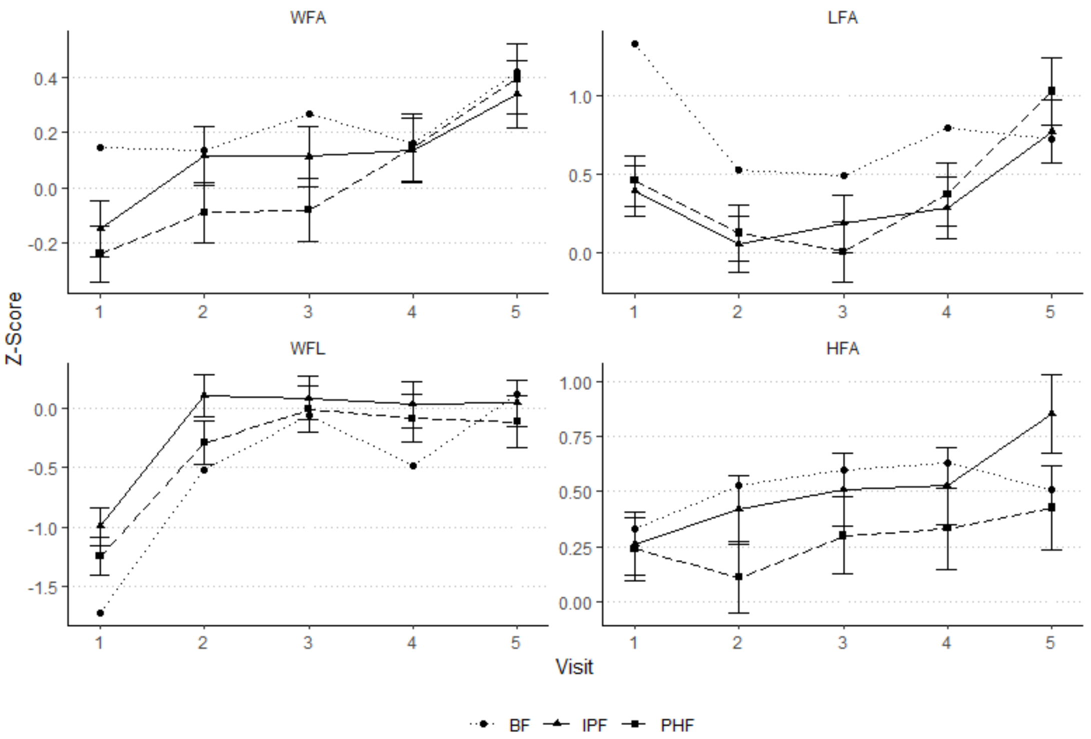

Figure S2. Z-Scores for weight-for-age (WFA), length-for-age (LFA), weight-for-length (WFL), and head circumference-for-age (HFA) for visits V1 to V5 for IPF and PHF in the ITT population in Germany. Each symbol represents the least square mean (LSM) +/- 1 x standard error of the mean (SEM). For reference, the breastfed group is shown with raw means. Based on WHO child growth standards [18].

IPF: infant formula manufactured from intact cow's milk proteins; ITT: intention-to-treat population; n: number of observations; PHF: infant formula manufactured from partially hydrolysed whey protein; WHO: World Health Organization.

V1: 0-28 days of age; V2: 30 ± 3 study days; V3: 60 ± 3 study days; V4: 90 ± 7 study days; V5: 6 months ± 7 study days.

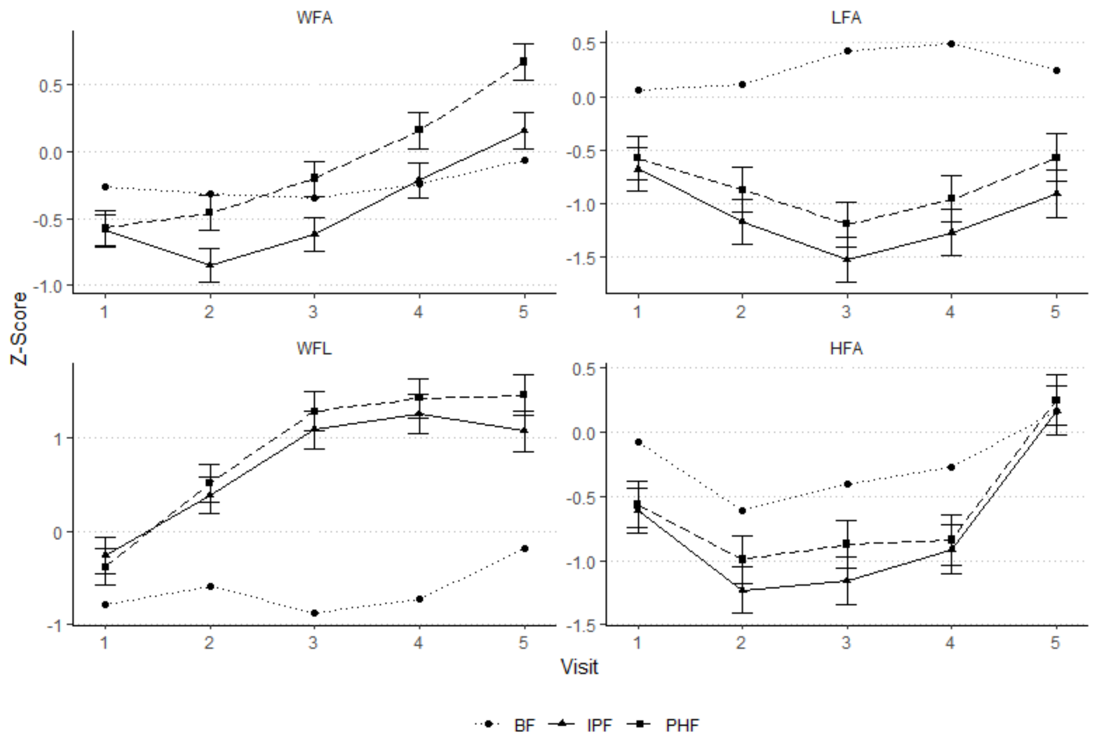

Figure S3. Z-Scores for weight-for-age (WFA), length-for-age (LFA), weight-for-length (WFL), and head circumference-for-age (HFA) for visits V1 to V5 for IPF and PHF in the ITT population in Bulgaria. Each symbol represents the least square mean (LSM) +/- 1 x standard error of the mean (SEM). For reference, the breastfed group is shown with raw means. Based on WHO child growth standards [18].

IPF: infant formula manufactured from intact cow's milk proteins; ITT: intention-to-treat population; n: number of observations; PHF: infant formula manufactured from partially hydrolysed whey protein; WHO: World Health Organization.

V1: 0-28 days of age; V2: 30 ± 3 study days; V3: 60 ± 3 study days; V4: 90 ± 7 study days; V5: 6 months ± 7 study days.

Table S2: Formula intake relative to body weight (ml/kg/day) in ITT to V5.

**Visit**	**IPF**			**PHF**			**p-value**
	**N**	**LSM**	**SEM**	**N**	**LSM**	**SEM**	
V2	90	159.5	3.08	81	161.4	3.24	0.677
V3	84	142.3	3.15	82	145.5	3.23	0.485
V4	85	128.9	3.15	74	130.3	3.34	0.761
V5	81	101.1	3.23	70	97.4	3.49	0.444

Values are presented as least-squares means (LSM) and standard error of the mean (SEM) for formula groups. Significant effect ($p<0.05$): gestational age and visit.

ANOVA: analysis of variance; IPF: infant formula manufactured from intact cow's milk proteins; ITT: intention-to-treat population; n: number of observations; PHF: infant formula manufactured from partially hydrolysed whey protein.

V1: 0-28 days of age; V2: 30 ± 3 study days; V3: 60 ± 3 study days; V4: 90 ± 7 study days; V5: 6 months ± 7 study days.
